# Supplementary material for: The Impact of Tokenizer Selection in Genomic Language Models
Source: bioRxiv. 2025 Jul 26:2024.09.09.612081. Preprint. [Version 3] doi: 10.1101/2024.09.09.612081 (PMC12330496; doi:10.1101/2024.09.09.612081)
Supplement: Supplement 1 [file media-1.pdf]

Supplemental Material: The Impact of Tokenizer Selection in Genomic Language Models

Acrynms

DNA - Deoxyribonucleic acid
NLP - Natural Language Processing
LMs - Language Models
pLMs - Protein Language Models
gLMs - Genomic Language Models
BPE - Byte Pair Encoding
OCR - Open Chromatin Region
CNN - Convolutional Neural Network
RNN - Recurrent Neural Network

Model Training Details

The CNN Baseline model used was the model specified in [5], as implemented in the Caduceus Github repository. For the GPT Baseline model we used EleutherAI/gpt-neo-125m, which is a EleutherAI's replication of the GPT-3 architecture. For DNABERT, and DNABERT-2, the pre-trained models provided by the authors were used (zhihan1996/DNA_bert_6, zhihan1996/DNABERT-2-117M). The InstaDeepAI/nucleotide-transformer-v2-500m-multi-species version of the Nucleotide Transformer was chosen as it was the largest multi-species model that would run on one Nvidia A100 GPU for fine-tuning. For the state space models, HyenaDNA, Mamba, and Caduceus, we pre-trained models using the pretraining script in the Caduceus github. Table 1 shows the model parameters that were chosen for each model. We have converted the perplexity values to a normalized unit of Bits per Character, which allows a more accurate comparison between models that use different tokenization methods.

We chose to use the Caduceus-ps (parameter-sharing) model to take full advantage of the new bi-mamba architecture that provides reverse compliment (RC) equivariance. The dimension parameter d was fixed at 256 to correspond with the other 4-layer pretrained models in the Caduceus huggingface repository.

We pretrained the state space models with an input data length of 4096 nucleotides. This length was chosen because there are no publicly available pretrained HyenaDNA or Caduceus models with sequence lengths between 1k and 16k nucleotides. This input length made the input range comparable to that of the DNABERT2 and Nucleotide Transformer models, minimizing the influence of the inherent long-range capabilities of the state space models. This strategy allowed us to isolate tokenization, as much as possible when comparing very different pretrained models, as an experimental variable.

We used a grid search method of hyperparameter tuning, varying only the batch size and the learning rates for 10,000 steps. After determining the best hyperparameters for pre-training, we then trained the best model another 10,000 steps. The number of steps was chosen based on reported results in [10] and [14].

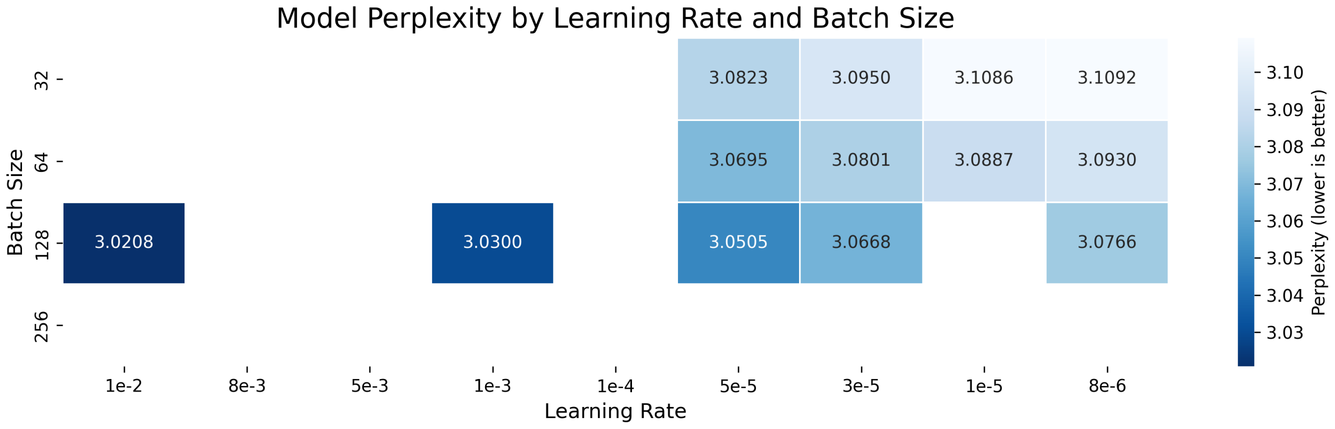

Fig. 1: Hyperparameter Tuning results for Pre-training the four layer Mamba model with byte-pair encoding. Missing cells are those that had NaN values. The model with the lowest perplexity after 10,000 steps was further trained until 20,000 steps and used as the model for fine-tuning. Similar plots were created for the other pretrained models

Table 1. Hyperparameter Tuning results for the models that we pretrained. Grid search hyperparameter tuning was completed for the first 10,000 steps of training and only the batch size and learning rate were varied. The model with the lowest perplexity was then further trained until 20,000 steps. The model parameters d, n, and k correspond to dimension, number of layers and input sequence length.

	Fixed Parameters				Hyperparameter Tuning		Best HP		Bits per Character
	d	n	k	RC Aug	Batch Size Range	Learning Rate Range	Batch Size	Learning Rate	Bits per Character
Mamba-char-4L	256	4	4096	TRUE	[128, 256]	[1e-2, 1e-3, 5e-3, 8e-3]	256	1e-2	1.545
Mamba-bpe-4L	256	4	4096	TRUE	[128, 256]	[1e-2, 8e-3, 5e-3, 1e-3, 1e-4, 5e-5, 3e-5, 1e-5, 8e-6]	128	1e-2	0.304
Caduceus-ps	256	4	4096	FALSE*	[128, 256]	[1e-3, 8e-3, 8e-4, 5e-4]	256	1e-3	1.374

*RC Aug (Reverse Compliment Augmentation) was not necesary for the Caduceus-ps (parameter sharing) model because the reverse compliment sequences are passed through one half of the bi-Mamba architecture. Reverse Compliment augmentation was used in the Mamba models to make sure that the architecture saw the same data as Caduceus-ps.

To verify that we had achieved the best performance for each downstream task, we conducted a comprehensive grid-based hyperparameter search for every downstream fine-tuning task. The results of these hyperparameter tuning experiments can be seen in Tables 2, 3, 4, 5, and 6.

We observed significant instability when using higher learning rates and small batch sizes, with a high occurance of a NaN loss. In order to determine the frequency of the NaN outputs, we performed a larger analysis on the GUE dataset using 6 different learning rates and 4 different batch sizes. The results of this analysis can be seen in the appendix in Figure 2. Higher learning rates were associated with higher instability, but also had the highest accuracy. Lower learning rates were much more stable but needed to be trained much longer to obtain the same level of accuracy. Although our experiments were conducted on the Mamba-bpe model, this instability has also been observed by several other users using the character tokenized Caduceus model, as reported in the Caduceus Github repository. This instability seems to be related to initial seed values and can be resolved by reducing the learning-rate and/or changing seed values.

Table 2. Best hyperparameters for Genomic Benchmark benchmark

Task	Model	LR	BS	MCC	Acc	N	MCC SD	Acc SD	LR Range	BS Range
demo_human_or_worm	Caduceus	5.0e-04	128	0.943	0.972	10	0.0014	0.0007	[1.0e-05, 1.0e-04, 5.0e-04, 1.0e-03, 2.0e-03]	[128, 256]
demo_human_or_worm	HyenaDNA	6.0e-05	256	0.929	0.964	13	0.0031	0.0016	[6.0e-05, 6.0e-04, 6.0e-03]	[128, 256]
demo_human_or_worm	Mamba-bpe	1.0e-04	256	0.923	0.961	13	0.0024	0.0013	[1.0e-05, 2.0e-05, 5.0e-05, 1.0e-04]	[128, 256]
demo_human_or_worm	Mamba-char	1.0e-03	128	0.930	0.965	13	0.0086	0.0043	[1.0e-04, 2.0e-04, 1.0e-03, 2.0e-03, 1.0e-02]	[128, 256]
dummy_mouse_enhancers	Caduceus	2.0e-03	128	0.583	0.788	10	0.0377	0.0210	[1.0e-05, 1.0e-04, 5.0e-04, 1.0e-03, 2.0e-03]	[128, 256]
dummy_mouse_enhancers	HyenaDNA	6.0e-04	128	0.595	0.795	14	0.0487	0.0261	[6.0e-05, 6.0e-04, 6.0e-03]	[128, 256]
dummy_mouse_enhancers	Mamba-bpe	5.0e-05	256	0.555	0.775	13	0.0325	0.0186	[1.0e-05, 5.0e-05, 1.0e-04]	[128, 256]
dummy_mouse_enhancers	Mamba-char	1.0e-03	128	0.570	0.785	13	0.0393	0.0199	[1.0e-03, 2.0e-03, 1.0e-02]	[128, 256]
human_enhancers_cohn	Caduceus	1.0e-03	128	0.493	0.746	10	0.0078	0.0041	[1.0e-05, 1.0e-04, 5.0e-04, 1.0e-03, 2.0e-03]	[128, 256]
human_enhancers_cohn	HyenaDNA	6.0e-05	128	0.469	0.734	13	0.0077	0.0039	[6.0e-05, 6.0e-04, 6.0e-03]	[128, 256]
human_enhancers_cohn	Mamba-bpe	1.0e-05	256	0.478	0.739	13	0.0044	0.0023	[1.0e-05, 2.0e-05, 5.0e-05, 1.0e-04]	[128, 256]
human_enhancers_cohn	Mamba-char	1.0e-03	256	0.473	0.735	13	0.0100	0.0054	[1.0e-04, 2.0e-04, 1.0e-03, 2.0e-03, 1.0e-02]	[128, 256]
human_enhancers_ensembl	Caduceus	1.0e-03	128	0.822	0.911	10	0.0052	0.0027	[1.0e-05, 1.0e-04, 5.0e-04, 1.0e-03, 2.0e-03]	[128, 256]
human_enhancers_ensembl	HyenaDNA	6.0e-04	256	0.757	0.878	13	0.0055	0.0029	[6.0e-05, 6.0e-04, 6.0e-03]	[128, 256]
human_enhancers_ensembl	Mamba-bpe	1.0e-04	128	0.728	0.862	14	0.0314	0.0162	[1.0e-05, 2.0e-05, 5.0e-05, 1.0e-04]	[128, 256]
human_enhancers_ensembl	Mamba-char	1.0e-03	256	0.743	0.871	13	0.0505	0.0260	[1.0e-04, 2.0e-04, 1.0e-03, 2.0e-03, 1.0e-02]	[128, 256]
human_ensembl_regulatory	Caduceus	5.0e-04	128	0.807	0.869	6	0.0081	0.0070	[1.0e-05, 1.0e-04, 5.0e-04, 1.0e-03, 2.0e-03]	[128, 256]
human_ensembl_regulatory	HyenaDNA	6.0e-05	128	0.795	0.861	13	0.0061	0.0049	[6.0e-05, 6.0e-04, 6.0e-03]	[128, 256]
human_ensembl_regulatory	Mamba-bpe	1.0e-05	128	0.839	0.892	10	0.0026	0.0018	[1.0e-05, 2.0e-05, 5.0e-05, 1.0e-04]	[128, 256]
human_ensembl_regulatory	Mamba-char	1.0e-03	256	0.605	0.733	13	0.1359	0.0924	[1.0e-04, 2.0e-04, 1.0e-03, 2.0e-03, 1.0e-02]	[128, 256]
human_nontata_promoters	Caduceus	1.0e-03	256	0.891	0.945	10	0.0033	0.0015	[1.0e-05, 1.0e-04, 5.0e-04, 1.0e-03, 2.0e-03]	[128, 256]
human_nontata_promoters	HyenaDNA	6.0e-04	256	0.880	0.940	13	0.0072	0.0036	[6.0e-05, 6.0e-04, 6.0e-03]	[128, 256]
human_nontata_promoters	Mamba-bpe	1.0e-04	256	0.895	0.947	13	0.0038	0.0019	[1.0e-05, 2.0e-05, 5.0e-05, 1.0e-04]	[128, 256]
human_nontata_promoters	Mamba-char	1.0e-03	256	0.911	0.955	13	0.0082	0.0043	[1.0e-04, 2.0e-04, 1.0e-03, 2.0e-03, 1.0e-02]	[128, 256]
human_ocr_ensembl	Caduceus	1.0e-03	128	0.635	0.817	7	0.0062	0.0033	[1.0e-05, 1.0e-04, 5.0e-04, 1.0e-03, 2.0e-03]	[128, 256]
human_ocr_ensembl	HyenaDNA	6.0e-04	256	0.605	0.802	13	0.0037	0.0020	[6.0e-05, 6.0e-04, 6.0e-03]	[128, 256]
human_ocr_ensembl	Mamba-bpe	5.0e-05	128	0.545	0.772	13	0.0052	0.0026	[1.0e-05, 2.0e-05, 5.0e-05, 1.0e-04]	[128, 256]
human_ocr_ensembl	Mamba-char	2.0e-03	256	0.632	0.816	13	0.0035	0.0017	[1.0e-04, 2.0e-04, 1.0e-03, 2.0e-03, 1.0e-02]	[128, 256]

Table 3. Best hyperparameters for GUE benchmark (Part 1 of 2)

Task	Model	LR	BS	MCC	Acc	N	MCC SD	Acc SD	LR Range	BS Range
covid	Caduceus	3.0e-04	128	0.630	0.672	4	0.0150	0.0135	[8.0e-05, 1.0e-04, 3.0e-04, 5.0e-04, 8.0e-04, 1.0e-03, 2.0e-03]	[128, 256, 512]
covid	HyenaDNA	6.0e-05	128	0.620	0.663	11	0.0205	0.0180	[6.0e-05, 6.0e-04, 6.0e-03]	[128, 256]
covid	Mamba-bpe	2.0e-04	128	0.652	0.691	13	0.0243	0.0232	[5.0e-05, 1.0e-04, 2.0e-04, 1.0e-03]	[128, 256]
covid	Mamba-char	2.0e-04	128	0.576	0.625	10	0.0110	0.0097	[5.0e-05, 1.0e-04, 2.0e-04]	[128, 256]
mouse_0	Caduceus	2.0e-03	128	0.610	0.803	10	0.0151	0.0080	[1.0e-05, 1.0e-04, 5.0e-04, 1.0e-03, 2.0e-03]	[128, 256]
mouse_0	HyenaDNA	6.0e-04	256	0.523	0.760	10	0.0220	0.0129	[6.0e-05, 6.0e-04, 6.0e-03]	[128, 256]
mouse_0	Mamba-bpe	1.0e-04	128	0.507	0.752	13	0.0161	0.0082	[1.0e-05, 2.0e-05, 5.0e-05, 1.0e-04, 2.0e-04, 6.0e-04]	[128, 256]
mouse_0	Mamba-char	2.0e-04	128	0.465	0.731	10	0.0185	0.0097	[5.0e-05, 1.0e-04, 2.0e-04]	[128, 256]
mouse_1	Caduceus	1.0e-03	128	0.845	0.923	10	0.0048	0.0025	[1.0e-05, 1.0e-04, 5.0e-04, 1.0e-03, 2.0e-03]	[128, 256]
mouse_1	HyenaDNA	6.0e-04	256	0.824	0.911	10	0.0036	0.0020	[6.0e-05, 6.0e-04, 6.0e-03]	[128, 256]
mouse_1	Mamba-bpe	2.0e-04	256	0.801	0.900	13	0.0055	0.0027	[1.0e-05, 2.0e-05, 5.0e-05, 1.0e-04, 2.0e-04]	[128, 256]
mouse_1	Mamba-char	2.0e-04	256	0.795	0.897	10	0.0035	0.0016	[5.0e-05, 1.0e-04, 2.0e-04]	[128, 256]
mouse_2	Caduceus	2.0e-03	128	0.845	0.921	7	0.0167	0.0081	[1.0e-05, 1.0e-04, 2.0e-04, 5.0e-04, 1.0e-03, 2.0e-03]	[128, 256]
mouse_2	HyenaDNA	6.0e-04	128	0.801	0.899	10	0.0320	0.0162	[6.0e-05, 6.0e-04, 6.0e-03]	[128, 256]
mouse_2	Mamba-bpe	2.0e-04	128	0.772	0.885	13	0.0212	0.0107	[1.0e-05, 2.0e-05, 5.0e-05, 1.0e-04, 2.0e-04, 2.0e-03]	[128, 256]
mouse_2	Mamba-char	2.0e-04	128	0.701	0.849	17	0.0274	0.0135	[1.0e-04, 2.0e-04]	[128, 256]
mouse_3	Caduceus	2.0e-03	128	0.827	0.911	10	0.0215	0.0108	[1.0e-05, 1.0e-04, 5.0e-04, 1.0e-03, 2.0e-03]	[128, 256]
mouse_3	HyenaDNA	6.0e-04	128	0.737	0.866	10	0.0477	0.0227	[6.0e-05, 6.0e-04, 6.0e-03]	[128, 256]
mouse_3	Mamba-bpe	2.0e-04	128	0.588	0.792	13	0.0471	0.0246	[1.0e-05, 2.0e-05, 5.0e-05, 1.0e-04, 2.0e-04, 2.0e-03]	[128, 256]
mouse_3	Mamba-char	2.0e-04	128	0.501	0.741	10	0.0283	0.0131	[5.0e-05, 1.0e-04, 2.0e-04]	[128, 256]
mouse_4	Caduceus	2.0e-03	256	0.496	0.746	10	0.0075	0.0045	[1.0e-05, 1.0e-04, 5.0e-04, 1.0e-03, 2.0e-03]	[128, 256]
mouse_4	HyenaDNA	6.0e-04	128	0.468	0.733	10	0.0159	0.0080	[6.0e-05, 6.0e-04, 6.0e-03]	[128, 256]
mouse_4	Mamba-bpe	5.0e-05	128	0.418	0.709	13	0.0128	0.0067	[1.0e-05, 2.0e-05, 5.0e-05, 1.0e-04, 2.0e-04]	[128, 256]
mouse_4	Mamba-char	2.0e-04	128	0.423	0.711	10	0.0165	0.0079	[5.0e-05, 1.0e-04, 2.0e-04]	[128, 256]
prom_300_all	Caduceus	5.0e-04	128	0.821	0.910	10	0.0054	0.0026	[1.0e-05, 1.0e-04, 5.0e-04, 1.0e-03, 2.0e-03]	[128, 256]
prom_300_all	HyenaDNA	6.0e-04	128	0.826	0.912	10	0.0052	0.0026	[6.0e-05, 6.0e-04, 6.0e-03]	[128, 256]
prom_300_all	Mamba-bpe	5.0e-05	256	0.810	0.904	13	0.0032	0.0016	[1.0e-05, 2.0e-05, 5.0e-05, 1.0e-04, 2.0e-04]	[128, 256]
prom_300_all	Mamba-char	1.0e-04	128	0.818	0.909	10	0.0065	0.0031	[5.0e-05, 1.0e-04, 2.0e-04]	[128, 256]
prom_300_notata	Caduceus	1.0e-03	128	0.928	0.964	10	0.0021	0.0011	[1.0e-05, 1.0e-04, 5.0e-04, 1.0e-03, 2.0e-03]	[128, 256]
prom_300_notata	HyenaDNA	6.0e-04	256	0.918	0.959	10	0.0054	0.0027	[6.0e-05, 6.0e-04, 6.0e-03]	[128, 256]
prom_300_notata	Mamba-bpe	5.0e-05	128	0.911	0.955	13	0.0046	0.0024	[1.0e-05, 2.0e-05, 5.0e-05, 1.0e-04, 2.0e-04]	[128, 256]
prom_300_notata	Mamba-char	2.0e-04	128	0.917	0.958	10	0.0027	0.0014	[5.0e-05, 1.0e-04, 2.0e-04]	[128, 256]
prom_300_tata	Caduceus	2.0e-03	128	0.581	0.789	10	0.0317	0.0162	[1.0e-05, 1.0e-04, 5.0e-04, 1.0e-03, 2.0e-03]	[128, 256]
prom_300_tata	HyenaDNA	6.0e-04	128	0.466	0.732	10	0.0431	0.0221	[6.0e-05, 6.0e-04, 6.0e-03]	[128, 256]
prom_300_tata	Mamba-bpe	5.0e-05	128	0.310	0.655	13	0.0330	0.0156	[1.0e-05, 2.0e-05, 5.0e-05, 1.0e-04, 2.0e-04]	[128, 256]
prom_300_tata	Mamba-char	2.0e-04	128	0.334	0.667	10	0.0300	0.0158	[5.0e-05, 1.0e-04, 2.0e-04]	[128, 256]
prom_core_all	Caduceus	1.0e-03	256	0.656	0.828	10	0.0085	0.0040	[1.0e-05, 1.0e-04, 5.0e-04, 1.0e-03, 2.0e-03]	[128, 256]

Table 4. Best hyperparameters for GUE benchmark (Part 2 of 2)

Task	Model	LR	BS	MCC	Acc	N	MCC SD	Acc SD	LR Range	BS Range
prom_core_all	HyenaDNA	6.0e-04	256	0.626	0.813	10	0.0077	0.0039	[6.0e-05, 6.0e-04, 6.0e-03]	[128, 256]
prom_core_all	Mamba-bpe	1.0e-04	128	0.596	0.798	13	0.0089	0.0044	[1.0e-05, 2.0e-05, 5.0e-05, 1.0e-04, 2.0e-04, 5.0e-04]	[128, 256]
prom_core_all	Mamba-char	2.0e-04	128	0.617	0.808	10	0.0069	0.0034	[5.0e-05, 1.0e-04, 2.0e-04]	[128, 256]
prom_core_notata	Caduceus	2.0e-03	256	0.675	0.836	10	0.0074	0.0047	[1.0e-05, 1.0e-04, 5.0e-04, 1.0e-03, 2.0e-03]	[128, 256]
prom_core_notata	HyenaDNA	6.0e-04	256	0.664	0.832	10	0.0036	0.0018	[6.0e-05, 6.0e-04, 6.0e-03]	[128, 256]
prom_core_notata	Mamba-bpe	5.0e-05	256	0.652	0.826	13	0.0066	0.0033	[1.0e-05, 2.0e-05, 5.0e-05, 1.0e-04, 2.0e-04]	[128, 256]
prom_core_notata	Mamba-char	2.0e-04	128	0.654	0.827	10	0.0042	0.0021	[5.0e-05, 1.0e-04, 2.0e-04]	[128, 256]
prom_core_tata	Caduceus	2.0e-03	128	0.595	0.794	10	0.1272	0.0675	[1.0e-05, 1.0e-04, 2.0e-04, 5.0e-04, 1.0e-03, 2.0e-03]	[128, 256]
prom_core_tata	HyenaDNA	6.0e-04	128	0.465	0.732	11	0.0314	0.0157	[6.0e-05, 6.0e-04, 6.0e-03]	[128, 256]
prom_core_tata	Mamba-bpe	2.0e-04	256	0.424	0.711	13	0.0279	0.0142	[1.0e-05, 2.0e-05, 5.0e-05, 1.0e-04, 2.0e-04]	[128, 256]
prom_core_tata	Mamba-char	2.0e-04	128	0.540	0.769	18	0.0177	0.0090	[5.0e-05, 1.0e-04, 2.0e-04]	[128, 256]
reconstructed	Caduceus	1.0e-03	256	0.816	0.893	10	0.0248	0.0144	[1.0e-04, 1.0e-03, 2.0e-03]	[128, 256]
reconstructed	HyenaDNA	6.0e-04	128	0.820	0.895	10	0.0119	0.0067	[6.0e-05, 6.0e-04, 6.0e-03]	[128, 256]
reconstructed	Mamba-bpe	2.0e-04	128	0.614	0.776	13	0.0157	0.0088	[5.0e-05, 1.0e-04, 2.0e-04]	[128, 256]
reconstructed	Mamba-char	2.0e-04	128	0.664	0.804	10	0.0249	0.0151	[5.0e-05, 1.0e-04, 2.0e-04]	[128, 256]
tf_0	Caduceus	5.0e-04	256	0.664	0.826	10	0.0134	0.0087	[1.0e-05, 1.0e-04, 5.0e-04, 1.0e-03, 2.0e-03]	[128, 256]
tf_0	HyenaDNA	6.0e-05	256	0.671	0.830	12	0.0082	0.0055	[6.0e-05, 6.0e-04, 6.0e-03]	[128, 256]
tf_0	Mamba-bpe	1.0e-04	256	0.643	0.815	13	0.0165	0.0100	[5.0e-05, 1.0e-04, 2.0e-04, 1.0e-03]	[128, 256]
tf_0	Mamba-char	2.0e-04	256	0.671	0.830	10	0.0128	0.0067	[5.0e-05, 1.0e-04, 2.0e-04]	[128, 256]
tf_1	Caduceus	1.0e-03	128	0.716	0.852	10	0.0180	0.0095	[1.0e-05, 1.0e-04, 5.0e-04, 1.0e-03, 2.0e-03]	[128, 256]
tf_1	HyenaDNA	6.0e-05	256	0.706	0.848	12	0.0123	0.0088	[6.0e-05, 6.0e-04, 6.0e-03]	[128, 256]
tf_1	Mamba-bpe	2.0e-04	128	0.683	0.836	12	0.0154	0.0107	[5.0e-05, 1.0e-04, 2.0e-04, 6.0e-04, 2.0e-03]	[128, 256]
tf_1	Mamba-char	5.0e-05	128	0.691	0.840	10	0.0094	0.0052	[5.0e-05, 1.0e-04, 2.0e-04]	[128, 256]
tf_2	Caduceus	1.0e-03	128	0.705	0.847	10	0.0331	0.0188	[1.0e-05, 1.0e-04, 5.0e-04, 1.0e-03, 2.0e-03]	[128, 256]
tf_2	HyenaDNA	6.0e-04	128	0.671	0.833	11	0.0224	0.0113	[6.0e-05, 6.0e-04, 6.0e-03]	[128, 256]
tf_2	Mamba-bpe	1.0e-04	128	0.571	0.782	13	0.0140	0.0067	[5.0e-05, 1.0e-04, 2.0e-04]	[128, 256]
tf_2	Mamba-char	2.0e-04	128	0.613	0.802	10	0.0243	0.0137	[5.0e-05, 1.0e-04, 2.0e-04]	[128, 256]
tf_3	Caduceus	2.0e-03	128	0.539	0.766	10	0.0544	0.0275	[1.0e-05, 1.0e-04, 5.0e-04, 1.0e-03, 2.0e-03]	[128, 256]
tf_3	HyenaDNA	6.0e-04	128	0.519	0.757	11	0.0195	0.0100	[6.0e-05, 6.0e-04, 6.0e-03]	[128, 256]
tf_3	Mamba-bpe	1.0e-04	128	0.480	0.736	13	0.0169	0.0110	[5.0e-05, 1.0e-04, 2.0e-04, 6.0e-04]	[128, 256]
tf_3	Mamba-char	1.0e-04	128	0.485	0.739	10	0.0217	0.0107	[5.0e-05, 1.0e-04, 2.0e-04]	[128, 256]
tf_4	Caduceus	2.0e-03	128	0.786	0.892	10	0.0307	0.0153	[1.0e-05, 1.0e-04, 5.0e-04, 1.0e-03, 2.0e-03]	[128, 256]
tf_4	HyenaDNA	6.0e-04	128	0.749	0.874	10	0.0158	0.0079	[6.0e-05, 6.0e-04, 6.0e-03]	[128, 256]
tf_4	Mamba-bpe	2.0e-04	128	0.697	0.848	13	0.0208	0.0104	[5.0e-05, 1.0e-04, 2.0e-04, 6.0e-04]	[128, 256]
tf_4	Mamba-char	1.0e-04	256	0.706	0.851	10	0.0060	0.0031	[5.0e-05, 1.0e-04, 2.0e-04]	[128, 256]

Table 5. Best hyperparameters for Nucleotide Transformer (revised) benchmark (Part 1 of 2)

Task	Model	LR	BS	MCC	Acc	N	MCC SD	Acc SD	LR Range	BS Range
H2AFZ	Caduceus	1.0e-04	128	0.432	0.708	7	0.0035	0.0012	[1.0e-05, 1.0e-04, 1.0e-03, 2.0e-03, 8.0e-03, 1.0e-02]	[128, 256]
H2AFZ	HyenaDNA	6.0e-04	128	0.413	0.702	13	0.0073	0.0035	[6.0e-05, 2.0e-04, 6.0e-04, 6.0e-03]	[128, 256]
H2AFZ	Mamba-bpe	5.0e-05	128	0.405	0.693	13	0.0128	0.0089	[5.0e-05, 1.0e-04, 2.0e-04]	[128, 256]
H2AFZ	Mamba-char	5.0e-04	128	0.392	0.688	18	0.0149	0.0107	[1.0e-04, 3.0e-04, 5.0e-04, 1.0e-03, 2.0e-03, 1.0e-02]	[128, 256]
H3K27ac	Caduceus	1.0e-03	128	0.467	0.733	3	0.0323	0.0162	[1.0e-05, 1.0e-04, 1.0e-03, 2.0e-03, 8.0e-03, 1.0e-02]	[128, 256]
H3K27ac	HyenaDNA	6.0e-05	256	0.421	0.707	13	0.0133	0.0078	[6.0e-05, 2.0e-04, 6.0e-04, 6.0e-03]	[128, 256]
H3K27ac	Mamba-bpe	5.0e-05	256	0.392	0.693	14	0.0145	0.0055	[5.0e-05, 1.0e-04, 2.0e-04]	[128, 256]
H3K27ac	Mamba-char	1.0e-04	256	0.418	0.705	18	0.0125	0.0101	[1.0e-04, 3.0e-04, 5.0e-04, 1.0e-03, 2.0e-03, 1.0e-02]	[128, 256]
H3K27me3	Caduceus	1.0e-03	128	0.551	0.763	3	0.0072	0.0047	[1.0e-05, 1.0e-04, 1.0e-03, 2.0e-03, 8.0e-03, 1.0e-02]	[128, 256]
H3K27me3	HyenaDNA	6.0e-05	256	0.542	0.764	13	0.0133	0.0078	[6.0e-05, 2.0e-04, 6.0e-04, 6.0e-03]	[128, 256]
H3K27me3	Mamba-bpe	5.0e-05	128	0.530	0.752	13	0.0096	0.0068	[5.0e-05, 1.0e-04, 2.0e-04]	[128, 256]
H3K27me3	Mamba-char	5.0e-04	128	0.535	0.757	18	0.0105	0.0083	[1.0e-04, 3.0e-04, 5.0e-04, 1.0e-03, 2.0e-03, 1.0e-02]	[128, 256]
H3K36me3	Caduceus	1.0e-03	256	0.531	0.760	3	0.0179	0.0076	[1.0e-05, 1.0e-04, 1.0e-03, 2.0e-03, 8.0e-03, 1.0e-02]	[128, 256]
H3K36me3	HyenaDNA	6.0e-04	256	0.504	0.746	13	0.0079	0.0059	[6.0e-05, 2.0e-04, 6.0e-04, 6.0e-03]	[128, 256]
H3K36me3	Mamba-bpe	5.0e-05	128	0.489	0.738	13	0.0096	0.0058	[5.0e-05, 1.0e-04, 2.0e-04, 3.0e-04]	[128, 256]
H3K36me3	Mamba-char	3.0e-04	128	0.486	0.737	18	0.0122	0.0088	[1.0e-04, 3.0e-04, 5.0e-04, 1.0e-03, 2.0e-03, 1.0e-02]	[128, 256]
H3K4me1	Caduceus	1.0e-03	128	0.429	0.706	11	0.0082	0.0101	[1.0e-05, 1.0e-04, 1.0e-03, 2.0e-03, 8.0e-03, 1.0e-02]	[128, 256]
H3K4me1	HyenaDNA	6.0e-04	128	0.403	0.695	11	0.0074	0.0041	[6.0e-05, 2.0e-04, 6.0e-04, 6.0e-03]	[128, 256]
H3K4me1	Mamba-bpe	5.0e-05	128	0.406	0.693	13	0.0095	0.0053	[5.0e-05, 1.0e-04, 2.0e-04]	[128, 256]
H3K4me1	Mamba-char	1.0e-04	128	0.405	0.692	18	0.0101	0.0054	[1.0e-04, 3.0e-04, 5.0e-04, 1.0e-03, 2.0e-03, 1.0e-02]	[128, 256]
H3K4me2	Caduceus	1.0e-03	128	0.485	0.736	7	0.0196	0.0121	[1.0e-05, 1.0e-04, 1.0e-03, 2.0e-03, 8.0e-03, 1.0e-02]	[128, 256]
H3K4me2	HyenaDNA	6.0e-04	128	0.477	0.732	13	0.0146	0.0091	[6.0e-05, 2.0e-04, 6.0e-04, 6.0e-03]	[128, 256]
H3K4me2	Mamba-bpe	5.0e-05	256	0.470	0.729	14	0.0144	0.0103	[5.0e-05, 1.0e-04, 2.0e-04]	[128, 256]
H3K4me2	Mamba-char	1.0e-04	256	0.456	0.724	18	0.0143	0.0080	[1.0e-04, 3.0e-04, 5.0e-04, 1.0e-03, 2.0e-03, 1.0e-02]	[128, 256]
H3K4me3	Caduceus	1.0e-04	128	0.623	0.811	10	0.0086	0.0045	[1.0e-05, 1.0e-04, 1.0e-03, 2.0e-03, 8.0e-03, 1.0e-02]	[128, 256]
H3K4me3	HyenaDNA	6.0e-04	256	0.600	0.799	11	0.0127	0.0057	[6.0e-05, 2.0e-04, 6.0e-04, 6.0e-03]	[128, 256]
H3K4me3	Mamba-bpe	5.0e-05	256	0.601	0.800	13	0.0158	0.0080	[5.0e-05, 1.0e-04, 2.0e-04]	[128, 256]
H3K4me3	Mamba-char	1.0e-04	256	0.606	0.802	18	0.0137	0.0073	[1.0e-04, 3.0e-04, 5.0e-04, 1.0e-03, 2.0e-03, 1.0e-02]	[128, 256]
H3K9ac	Caduceus	1.0e-04	256	0.512	0.755	3	0.0039	0.0023	[1.0e-05, 1.0e-04, 1.0e-03, 2.0e-03, 8.0e-03, 1.0e-02]	[128, 256]
H3K9ac	HyenaDNA	2.0e-04	256	0.505	0.751	12	0.0117	0.0068	[6.0e-05, 2.0e-04, 6.0e-04, 6.0e-03]	[128, 256]
H3K9ac	Mamba-bpe	5.0e-05	128	0.477	0.737	13	0.0212	0.0103	[5.0e-05, 1.0e-04, 2.0e-04]	[128, 256]
H3K9ac	Mamba-char	5.0e-04	128	0.502	0.749	18	0.0193	0.0101	[1.0e-04, 3.0e-04, 5.0e-04, 1.0e-03, 2.0e-03, 1.0e-02]	[128, 256]
H3K9me3	Caduceus	1.0e-03	256	0.395	0.697	3	0.0215	0.0105	[1.0e-05, 1.0e-04, 1.0e-03, 2.0e-03, 8.0e-03, 1.0e-02]	[128, 256]
H3K9me3	HyenaDNA	6.0e-04	256	0.370	0.681	13	0.0308	0.0164	[6.0e-05, 2.0e-04, 6.0e-04, 6.0e-03]	[128, 256]
H3K9me3	Mamba-bpe	1.0e-04	128	0.290	0.643	13	0.0285	0.0144	[5.0e-05, 1.0e-04, 2.0e-04]	[128, 256]
H3K9me3	Mamba-char	3.0e-04	128	0.340	0.668	18	0.0168	0.0089	[1.0e-04, 3.0e-04, 5.0e-04, 1.0e-03, 2.0e-03, 1.0e-02]	[128, 256]
H4K20me1	Caduceus	1.0e-03	128	0.589	0.788	3	0.0059	0.0024	[1.0e-05, 1.0e-04, 1.0e-03, 2.0e-03, 8.0e-03, 1.0e-02]	[128, 256]

Table 6. Best hyperparameters for Nucleotide Transformer (revised) benchmark (Part 2 of 2)

Task	Model	LR	BS	MCC	Acc	N	MCC SD	Acc SD	LR Range	BS Range
H4K20me1	HyenaDNA	6.0e-05	256	0.574	0.782	13	0.0137	0.0086	[6.0e-05, 2.0e-04, 6.0e-04, 6.0e-03]	[128, 256]
H4K20me1	Mamba-bpe	5.0e-05	256	0.544	0.766	14	0.0090	0.0066	[5.0e-05, 1.0e-04, 2.0e-04]	[128, 256]
H4K20me1	Mamba-char	5.0e-04	256	0.560	0.775	18	0.0105	0.0085	[1.0e-04, 3.0e-04, 5.0e-04, 1.0e-03, 2.0e-03, 1.0e-02]	[128, 256]
enhancers	Caduceus	1.0e-03	128	0.505	0.745	10	0.0083	0.0082	[1.0e-05, 1.0e-04, 1.0e-03, 2.0e-03, 8.0e-03, 1.0e-02]	[128, 256]
enhancers	HyenaDNA	2.0e-04	256	0.492	0.740	13	0.0108	0.0044	[6.0e-05, 2.0e-04, 6.0e-04, 6.0e-03]	[128, 256]
enhancers	Mamba-bpe	5.0e-05	256	0.487	0.736	13	0.0079	0.0057	[5.0e-05, 1.0e-04, 2.0e-04]	[128, 256]
enhancers	Mamba-char	2.0e-03	256	0.489	0.737	13	0.0111	0.0049	[1.0e-03, 2.0e-03, 1.0e-02]	[128, 256]
enhancers_types	Caduceus	1.0e-03	128	0.461	0.709	10	0.0101	0.0110	[1.0e-05, 1.0e-04, 1.0e-03, 2.0e-03, 8.0e-03, 1.0e-02]	[128, 256]
enhancers_types	HyenaDNA	2.0e-04	256	0.447	0.703	11	0.0114	0.0065	[6.0e-05, 2.0e-04, 6.0e-04, 6.0e-03]	[128, 256]
enhancers_types	Mamba-bpe	5.0e-05	256	0.449	0.700	13	0.0101	0.0056	[5.0e-05, 1.0e-04, 2.0e-04]	[128, 256]
enhancers_types	Mamba-char	2.0e-03	128	0.462	0.709	18	0.0091	0.0075	[1.0e-03, 2.0e-03, 1.0e-02]	[128, 256]
promoter_all	Caduceus	1.0e-05	128	0.718	0.859	10	0.0045	0.0021	[1.0e-05, 1.0e-04, 1.0e-03, 2.0e-03, 8.0e-03, 1.0e-02]	[128, 256]
promoter_all	HyenaDNA	6.0e-05	128	0.708	0.854	11	0.0102	0.0051	[6.0e-05, 2.0e-04, 6.0e-04, 6.0e-03]	[128, 256]
promoter_all	Mamba-bpe	2.0e-04	128	0.698	0.848	13	0.0112	0.0056	[5.0e-05, 1.0e-04, 2.0e-04]	[128, 256]
promoter_all	Mamba-char	2.0e-03	128	0.711	0.854	13	0.0086	0.0043	[1.0e-03, 2.0e-03, 1.0e-02]	[128, 256]
promoter_no_tata	Caduceus	1.0e-04	128	0.738	0.868	10	0.0081	0.0046	[1.0e-05, 1.0e-04, 1.0e-03, 2.0e-03, 8.0e-03, 1.0e-02]	[128, 256]
promoter_no_tata	HyenaDNA	6.0e-05	256	0.735	0.867	13	0.0052	0.0030	[6.0e-05, 2.0e-04, 6.0e-04, 6.0e-03]	[128, 256]
promoter_no_tata	Mamba-bpe	5.0e-05	256	0.727	0.863	13	0.0086	0.0043	[5.0e-05, 1.0e-04, 2.0e-04]	[128, 256]
promoter_no_tata	Mamba-char	2.0e-03	128	0.733	0.866	13	0.0097	0.0048	[1.0e-03, 2.0e-03, 1.0e-02]	[128, 256]
promoter_tata	Caduceus	1.0e-03	128	0.838	0.919	10	0.0347	0.0173	[1.0e-05, 1.0e-04, 1.0e-03, 2.0e-03, 8.0e-03, 1.0e-02]	[128, 256]
promoter_tata	HyenaDNA	2.0e-04	128	0.671	0.834	11	0.0328	0.0164	[6.0e-05, 2.0e-04, 6.0e-04, 6.0e-03]	[128, 256]
promoter_tata	Mamba-bpe	2.0e-04	256	0.621	0.808	13	0.0318	0.0168	[5.0e-05, 1.0e-04, 2.0e-04]	[128, 256]
promoter_tata	Mamba-char	1.0e-03	128	0.676	0.836	13	0.0403	0.0207	[1.0e-03, 2.0e-03, 1.0e-02]	[128, 256]
splice_sites_acceptors	Caduceus	1.0e-03	128	0.873	0.937	10	0.0090	0.0045	[1.0e-05, 1.0e-04, 1.0e-03, 2.0e-03, 8.0e-03, 1.0e-02]	[128, 256]
splice_sites_acceptors	HyenaDNA	6.0e-04	128	0.887	0.944	13	0.0164	0.0081	[6.0e-05, 2.0e-04, 6.0e-04, 6.0e-03]	[128, 256]
splice_sites_acceptors	Mamba-bpe	2.0e-04	128	0.607	0.802	13	0.0290	0.0159	[5.0e-05, 1.0e-04, 2.0e-04]	[128, 256]
splice_sites_acceptors	Mamba-char	2.0e-03	128	0.902	0.951	13	0.0072	0.0036	[1.0e-03, 2.0e-03, 1.0e-02]	[128, 256]
splice_sites_all	Caduceus	1.0e-03	128	0.911	0.941	10	0.0102	0.0068	[1.0e-05, 1.0e-04, 1.0e-03, 2.0e-03, 8.0e-03, 1.0e-02]	[128, 256]
splice_sites_all	HyenaDNA	6.0e-04	128	0.909	0.939	13	0.0155	0.0103	[6.0e-05, 2.0e-04, 6.0e-04, 6.0e-03]	[128, 256]
splice_sites_all	Mamba-bpe	2.0e-04	128	0.705	0.803	13	0.0126	0.0089	[5.0e-05, 1.0e-04, 2.0e-04]	[128, 256]
splice_sites_all	Mamba-char	2.0e-03	128	0.930	0.953	13	0.0057	0.0039	[1.0e-03, 2.0e-03, 1.0e-02]	[128, 256]
splice_sites_donors	Caduceus	1.0e-03	256	0.880	0.940	3	0.0164	0.0082	[1.0e-05, 1.0e-04, 1.0e-03, 2.0e-03, 8.0e-03, 1.0e-02]	[128, 256]
splice_sites_donors	HyenaDNA	6.0e-04	128	0.919	0.960	13	0.0305	0.0153	[6.0e-05, 2.0e-04, 6.0e-04, 6.0e-03]	[128, 256]
splice_sites_donors	Mamba-bpe	2.0e-04	128	0.598	0.797	13	0.0173	0.0096	[5.0e-05, 1.0e-04, 2.0e-04]	[128, 256]
splice_sites_donors	Mamba-char	2.0e-03	128	0.919	0.960	18	0.0534	0.0268	[1.0e-03, 2.0e-03, 1.0e-02]	[128, 256]

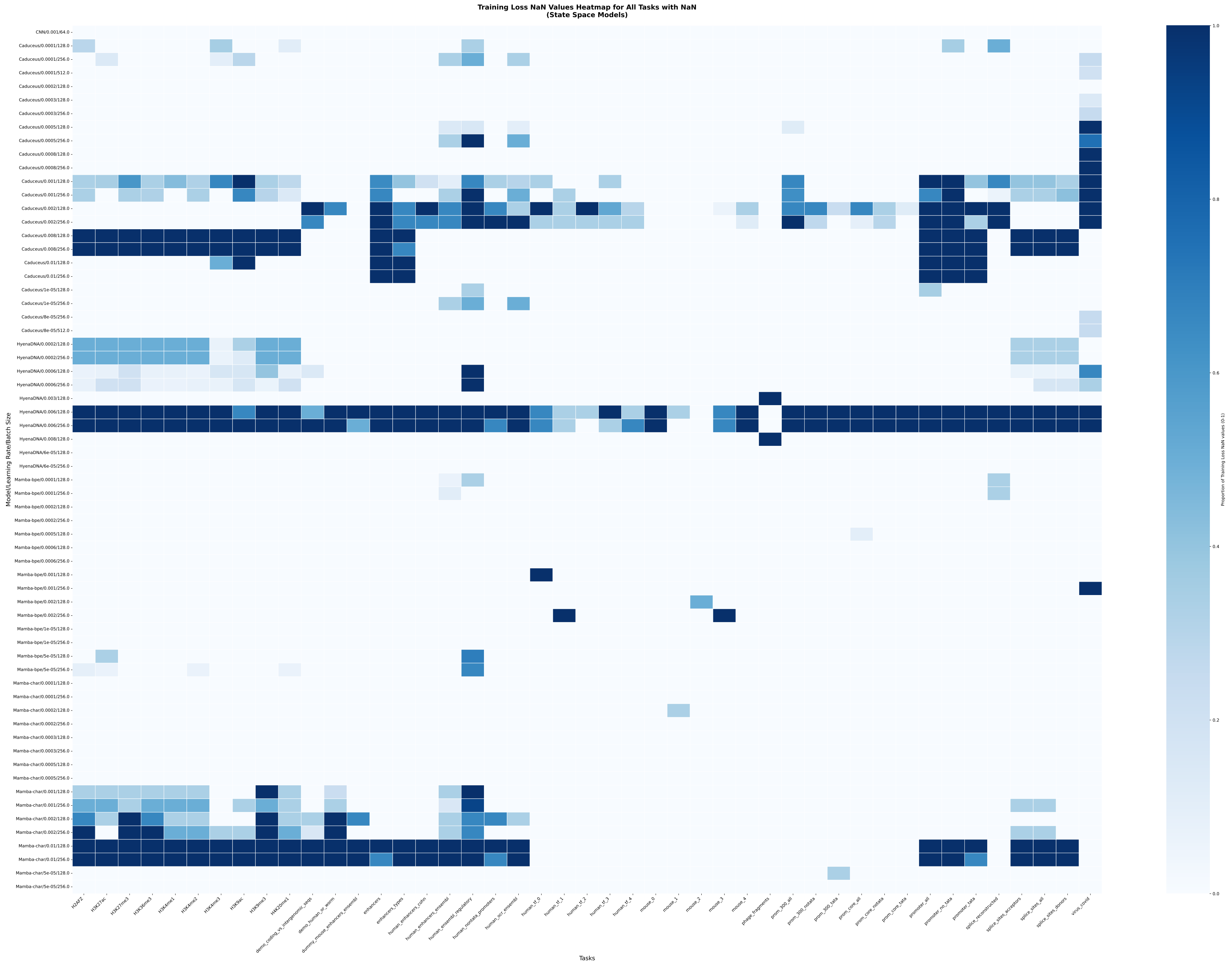

Fig. 2: Training Instability by learning rate and batch size. This heatmap indicates the percentage of instances that resulted in unstable fine-tuning on the genomic benchmark, depending upon the learning rate and batch sizes chosen. Instability is defined here as the number of times that training resulted in a training loss that overflows the precision of the machine and results in a loss of NaN. This typically happens when there is a gradient explosion during training.

Complete Benchmarking Results

Accuracy and MCC scores on the Genomic Benchmark can be seen in Tables 7 and 8. The results for the Genome Understanding Evaluation benchmark can be seen in Tables 9 and 10, and the results for the Nucleotide Transformer tasks can be seen in Tables 11 and 12.

Table 7. Accuracy Scores on the Genomic Benchmark. The highest score for each dataset is highlighted in bold and underlined.

	**CNN (char)**	**GPT-2 (bpe)**	**DNABERT2 (bpe)**	**Mamba (bpe)**	**NT (blocked k-mer)**	**DNABERT (k-mer)**	**HyenaDNA (char)**	**Mamba (char)**	**Caduceus (char)**
Mouse Enhancers	0.715	0.679	0.716	0.775	0.693	0.630	**0.795**	0.785	0.788
Coding vs. Intergenic	0.892	0.827	**0.948**	0.896	0.903	0.867	0.901	0.883	0.914
Human vs. Worm	0.942	0.917	**0.974**	0.961	0.932	0.910	0.964	0.965	0.972
Enhancers Cohn	0.702	0.682	0.746	0.739	0.682	0.652	0.734	0.735	**0.746**
Enhancers Ensembl	0.767	0.815	0.902	0.862	0.834	0.804	0.878	0.871	**0.911**
Ensembl Regulatory	0.866	0.883	0.771	0.892	**0.937**	0.771	0.861	0.733	0.869
Non-Tata Promoters	0.860	0.847	**0.960**	0.947	0.834	0.784	0.940	0.955	0.945
OCR Ensembl	0.695	0.634	0.812	0.772	0.686	0.631	0.802	0.816	**0.817**
	Baseline		*Sub-word Tokenization*			*Nucleotide Level Tokenization*			

Table 8. MCC Scores on the Genomic Benchmark. The highest score for each dataset is highlighted in bold and underlined.

	**CNN (char)**	**GPT-2 (bpe)**	**DNABERT2 (bpe)**	**Mamba (bpe)**	**NT (blocked k-mer)**	**DNABERT (k-mer)**	**HyenaDNA (char)**	**Mamba (char)**	**Caduceus (char)**
Mouse Enhancers	0.433	0.367	0.482	0.555	0.390	0.257	**0.595**	0.570	0.583
Coding vs. Intergenic	0.784	0.659	**0.896**	0.793	0.807	0.733	0.803	0.774	0.828
Human vs. Worm	0.884	0.836	**0.949**	0.923	0.865	0.820	0.929	0.930	0.943
Enhancers Cohn	0.409	0.365	0.493	0.478	0.368	0.305	0.469	0.473	**0.493**
Enhancers Ensembl	0.549	0.631	0.805	0.728	0.669	0.609	0.757	0.743	**0.822**
Ensembl Regulatory	0.801	0.730	0.447	0.839	**0.856**	0.445	0.795	0.605	0.807
Non-Tata Promoters	0.721	0.703	**0.920**	0.895	0.673	0.586	0.880	0.911	0.891
OCR Ensembl	0.393	0.270	0.625	0.545	0.373	0.263	0.605	0.632	**0.635**
	Baseline		*Sub-word Tokenization*			*Nucleotide Level Tokenization*			

Table 9. Accuracy Scores on the GUE. The highest score for each dataset is highlighted in bold and underlined.

	**CNN (char)**	**GPT-2 (bpe)**	**DNABERT2 (bpe)**	**Mamba (bpe)**	**NT (blocked k-mer)**	**DNABERT (k-mer)**	**HyenaDNA (char)**	**Mamba (char)**	**Caduceus (char)**
prom_core_all	0.808	0.815	0.816	0.798	0.771	**0.853**	0.813	0.808	0.828
prom_core_notata	0.807	0.825	0.840	0.826	0.788	**0.854**	0.832	0.827	0.836
prom_core_tata	0.879	0.832	0.870	0.711	0.860	**0.882**	0.732	0.769	0.794
prom_300_all	0.903	0.899	0.923	0.904	0.908	**0.955**	0.912	0.909	0.910
prom_300_notata	0.936	0.944	**0.966**	0.955	0.927	0.964	0.959	0.958	0.964
prom_300_tata	0.856	0.789	0.823	0.655	**0.901**	0.836	0.732	0.667	0.789
human_tfp_0	0.784	0.821	0.829	0.815	0.777	0.830	**0.830**	0.830	0.826
human_tfp_1	0.818	0.833	**0.856**	0.836	0.799	0.849	0.848	0.840	0.852
human_tfp_2	0.767	0.761	0.825	0.782	0.719	0.823	0.833	0.802	**0.847**
human_tfp_3	0.678	0.686	**0.790**	0.736	0.649	0.773	0.757	0.739	0.766
human_tfp_4	0.815	0.826	0.880	0.848	0.785	0.867	0.874	0.851	**0.892**
mouse_tfp_0	0.645	0.662	0.797	0.752	0.669	0.726	0.760	0.731	**0.803**
mouse_tfp_1	0.818	0.882	0.906	0.900	0.864	0.898	0.911	0.897	**0.923**
mouse_tfp_2	0.786	0.865	0.914	0.885	0.840	0.862	0.899	0.849	**0.921**
mouse_tfp_3	0.688	0.760	0.880	0.792	0.712	0.826	0.866	0.741	**0.911**
mouse_tfp_4	0.654	0.659	0.730	0.709	0.609	0.707	0.733	0.711	**0.746**
virus_covid	0.227	0.601	0.470	**0.691**	0.555	0.456	0.663	0.625	0.672
splice_reconstructed	0.862	0.835	0.910	0.776	**0.913**	0.906	0.895	0.804	0.893
	Baseline		*Sub-word Tokenization*			*Nucleotide Level Tokenization*			

Table 10. MCC Scores on the GUE. The highest score for each dataset is highlighted in bold and underlined.

	CNN (char)	GPT-2 (bpe)	DNABERT2 (bpe)	Mamba (bpe)	NT (blocked k-mer)	DNABERT (k-mer)	HyenaDNA (char)	Mamba (char)	Caduceus (char)
prom_core_all	0.617	0.630	0.633	0.596	0.548	**0.708**	0.626	0.617	0.656
prom_core_notata	0.616	0.650	0.680	0.652	0.576	**0.708**	0.664	0.654	0.675
prom_core_tata	0.761	0.670	0.744	0.424	0.722	**0.764**	0.465	0.540	0.595
prom_300_all	0.807	0.798	0.847	0.810	0.816	**0.909**	0.826	0.818	0.821
prom_300_notata	0.873	0.888	**0.932**	0.911	0.855	0.929	0.918	0.917	0.928
prom_300_tata	0.712	0.579	0.647	0.310	**0.804**	0.675	0.466	0.334	0.581
human_tfp_0	0.582	0.650	0.668	0.643	0.567	0.666	**0.671**	0.671	0.664
human_tfp_1	0.643	0.674	**0.718**	0.683	0.605	0.703	0.706	0.691	0.716
human_tfp_2	0.542	0.527	0.652	0.571	0.446	0.654	0.671	0.613	**0.705**
human_tfp_3	0.357	0.383	**0.587**	0.480	0.309	0.551	0.519	0.485	0.539
human_tfp_4	0.639	0.656	0.764	0.697	0.573	0.739	0.749	0.706	**0.786**
mouse_tfp_0	0.294	0.331	0.596	0.507	0.340	0.452	0.523	0.465	**0.610**
mouse_tfp_1	0.637	0.765	0.812	0.801	0.728	0.797	0.824	0.795	**0.845**
mouse_tfp_2	0.574	0.731	0.829	0.772	0.682	0.726	0.801	0.701	**0.845**
mouse_tfp_3	0.385	0.530	0.760	0.588	0.432	0.653	0.737	0.501	**0.827**
mouse_tfp_4	0.308	0.319	0.462	0.418	0.218	0.414	0.468	0.423	**0.496**
virus_covid	0.128	0.549	0.401	**0.652**	0.498	0.389	0.620	0.576	0.630
splice_reconstructed	0.766	0.718	0.846	0.614	**0.852**	0.840	0.820	0.664	0.816
	Baseline		*Sub-word Tokenization*			*Nucleotide Level Tokenization*			

Table 11. Accuracy Scores on the Nucleotide Transformer (revised). The highest score for each dataset is highlighted in bold and underlined.

	CNN (char)	GPT-2 (bpe)	DNABERT2 (bpe)	Mamba (bpe)	NT (blocked k-mer)	DNABERT (k-mer)	HyenaDNA (char)	Mamba (char)	Caduceus (char)
H2AFZ	0.723	0.737	0.756	0.693	**0.761**	0.747	0.702	0.688	0.708
H3K27ac	0.704	0.722	0.746	0.693	**0.751**	0.724	0.707	0.705	0.733
H3K27me3	0.746	0.771	0.799	0.752	**0.801**	0.774	0.764	0.757	0.763
H3K36me3	0.762	0.762	0.811	0.738	**0.828**	0.778	0.746	0.737	0.760
H3K4me1	0.690	0.711	0.749	0.693	**0.752**	0.727	0.695	0.692	0.706
H3K4me2	0.743	0.758	**0.782**	0.729	0.777	0.763	0.732	0.724	0.736
H3K4me3	0.789	0.790	0.816	0.800	**0.822**	0.808	0.799	0.802	0.811
H3K9ac	0.751	0.746	0.776	0.737	**0.778**	0.750	0.751	0.749	0.755
H3K9me3	0.666	0.691	0.746	0.643	**0.749**	0.714	0.681	0.668	0.697
H4K20me1	0.776	0.791	0.821	0.766	**0.830**	0.800	0.782	0.775	0.788
promoter_all	0.867	0.852	0.878	0.848	**0.892**	0.864	0.854	0.854	0.859
promoter_no_tata	0.864	0.869	0.880	0.863	**0.896**	0.869	0.867	0.866	0.868
promoter_tata	**0.943**	0.890	0.928	0.808	0.942	0.917	0.834	0.836	0.919
enhancers	0.745	0.731	0.755	0.736	**0.797**	0.761	0.740	0.737	0.745
enhancers_types	0.705	0.698	0.727	0.700	**0.771**	0.728	0.703	0.709	0.709
splice_site_prediction	0.951	0.831	0.903	0.803	**0.978**	0.974	0.939	0.953	0.941
splice_sites_acceptors	0.964	0.868	0.904	0.802	**0.985**	0.979	0.944	0.951	0.937
splice_sites_donors	0.976	0.874	0.915	0.797	**0.986**	0.983	0.960	0.960	0.940
	Baseline		*Sub-word Tokenization*			*Nucleotide Level Tokenization*			

Genome Benchmark

The Genomic Benchmark was introduced by Gresova et al [5] in 2023. This benchmark includes identification of regulatory elements in human DNA, discriminating between coding and non-coding DNA, identification of regulatory elements in mouse and drosophilia DNA and discriminating between Human and Worm DNA.

Human Enhancers Cohn This dataset was adapted from Cohn et al. 2018 [2]. This consists of simulated sequences, in vivo binding data for individual transcription factors, and genome-wide chromatin maps of active enhancers.

Human Enhancers Ensembl This dataset includes human enhancers from The FANTOM5 project by Andersson et al. 2014 [1]. The human genome GRCh38 was used to randomly generate negative sequences that match the lengths of the positive sequences without overlap.

Human Ensemble Regulatory This dataset was retrieved from Howe et al. 2021 [6] which contains human enhancer, promoter, and open chromatin region sequences from Zerbino et al 2015 [13].

Human Non-tata Promoters This dataset was curated by Umarov et al. [12] and consists of promoters from human, mouse, plant (Arabidopsis), and the bacteria Escherichia coli and Bacillus subtilis. E. coli promoters were extracted from RegulonDB and Bacillus subtilus promoters were taken from DBTBS [7]. Both positive and negative examples were 81 nucleotides in length. The negative samples

Table 12. MCC Scores on the Nucleotide Transformer (revised). The highest score for each dataset is highlighted in bold and underlined.

	CNN (char)	GPT-2 (bpe)	DNABERT2 (bpe)	Mamba (bpe)	NT (blocked k-mer)	DNABERT (k-mer)	HyenaDNA (char)	Mamba (char)	Caduceus (char)
H2AFZ	0.455	0.483	0.525	0.405	**0.526**	0.504	0.413	0.392	0.432
H3K27ac	0.409	0.445	0.500	0.392	**0.504**	0.455	0.421	0.418	0.467
H3K27me3	0.509	0.555	0.609	0.530	**0.619**	0.568	0.542	0.535	0.551
H3K36me3	0.540	0.539	0.627	0.489	**0.662**	0.572	0.504	0.486	0.531
H3K4me1	0.406	0.431	0.506	0.406	**0.513**	0.467	0.403	0.405	0.429
H3K4me2	0.487	0.534	**0.575**	0.470	0.562	0.536	0.477	0.456	0.485
H3K4me3	0.581	0.581	0.634	0.601	**0.644**	0.616	0.600	0.606	0.623
H3K9ac	0.503	0.495	0.555	0.477	**0.556**	0.504	0.505	0.502	0.512
H3K9me3	0.344	0.388	0.495	0.290	**0.499**	0.433	0.370	0.340	0.395
H4K20me1	0.560	0.588	0.657	0.544	**0.674**	0.609	0.574	0.560	0.589
promoter_all	0.735	0.704	0.758	0.698	**0.785**	0.728	0.708	0.711	0.718
promoter_no_tata	0.731	0.738	0.761	0.727	**0.793**	0.740	0.735	0.733	0.738
promoter_tata	**0.888**	0.784	0.858	0.621	0.885	0.834	0.671	0.676	0.838
enhancers	0.501	0.475	0.523	0.487	**0.599**	0.539	0.492	0.489	0.505
enhancers_types	0.442	0.447	0.493	0.449	**0.572**	0.495	0.447	0.462	0.461
splice_site_prediction	0.927	0.748	0.854	0.705	**0.967**	0.961	0.909	0.930	0.911
splice_sites_acceptors	0.928	0.736	0.808	0.607	**0.971**	0.957	0.887	0.902	0.873
splice_sites_donors	0.953	0.750	0.829	0.598	**0.973**	0.967	0.919	0.919	0.880
	Baseline		*Sub-word Tokenization*			*Nucleotide Level Tokenization*			

were generated by randomly selecting fragments of protein-coding genes and then taking the reverse complement of the strand. Eukaryotic promoters were extracted from EPD [4]. Negative examples were randomly generated segments of length 251 from the gene located after the first exon.

Human OCR Ensembl This dataset was adapted from Howe et a. 2021 [6] and Zerbino et al. 2015 [13]. The positive examples in this dataset are human open chromatin regions that were seen in experiments using DNase-seq but are not included in any other categories like enhancer, promoter, gene, TSS, or CTCF [13]. Using the human genome GRCh38, negative examples were randomly generated to match the lengths of the positive sequences without overlap.

Human or Worm (demo) This is a demo dataset compiled by Gresova et al. [5]. It consists of randomly selected 200 base pair long human and C. elegans genomic sequences with a 75:25 train-test split.

Coding or Intergenomic (demo) This is a dataset compiled of randomly selected 200 base pair intervals from intergenomic regions or protein coding transcipts of the human genome with a 75:25 train-test split. The datasets are from the simecek/PseudoDNA_Generator GitHub repo [11] and were used in the ECCB deep learning workshop.

Mouse Enhancers Ensembl (dummy) The dataset was curated using data from Ensembl release 100. Positive sequences consist of mouse enhancers sourced from the VISTA Enhancer Browser, while negative sequences were randomly generated from the mouse genome GRCm38 to match the lengths of the positive sequences without overlapping.

Nucleotide Transformer Tasks (revised)

A set of genomic classification tasks was introduced by Dalle-Torre et al [3]. This version of the benchmark supercedes the original version. It contains ten binary classification tasks of epigenetic mark prediction from a human dataset, a dataset of regulatory elements from multiple species and a splice site detection dataset from human DNA. In all there are 18 fine-tuning tasks with either two or three labeled classes.

Promoter Detection (Human) This dataset was constructed by the authors by downloading all human promoters from the Eukaryotic Promoter Database [9], selecting the region that spans 49 nt upstream and 10 nt downstream from the transcription start sites. They then selected 300-nt regions that contained promoters as positive examples and all remaining 300-nt regions as negative examples. These were then split to create three different tasks: the presence of any promoter element (promoter all), a promoter with a tata box or a promoter without a tata box.

Enhancers (Human) Human enhancer elements were downloaded from the ENCODE's SCREEN database [8] and and split into tissue-specific or tissue-invariant. Regions 400 nt in length containing enhancers were selected as positive and not overlapping enhancers were labeled negative. The enhancer task is a binary classification task, with the model identifying a segment as containing or not containing an enhancer. The enhancer types dataset is a multi-label prediction task with the three labels: tissue-specific enhancer, tissue-invariant enhancer or none.

Splice Site Prediction (Human) Human annotated splice sites were downloaded from GENCODE and filtered to exclude level-3 transcripts to obtain the highest quality training dataset. Splice site annotations were extracted and then 600 nt genomic regions were selected with the positive set containing a splice donor or acceptor site in the center of the window and all remaining 600 nt segments that were not overlapping a splice site were labeled as negative. Three different tasks were created from this dataset, a binary classification task for donors, acceptors and a multi-label prediction task with the labels being acceptor, donor or none.

Epigenetic Marks Prediction (Human) This data was curated by the authors by downloading histone ChIP-seq data for ten histone marks in the K562 human cell line from ENCODE [8] A 1-kb genomic sequence containing peaks were selected for each positive example and all 1-kb sequences that were not overlapping any peaks were selected as negative examples.

Dataset splits and subsampling Each dataset was balanced by subsampling the negative examles to the same number as positive examples. To reduce the overal size of the datasets, the datasets were further randomly sampled to create a training dataset of size 30000 and a validation and test dataset of size 3000.

Genome Understanding Evaluation

The authors of DNABERT-2 introduced a new Genomic Understanding Evaluation (GUE) Benchmark in 2023 [15]. They felt that previously published genomic benchmarks were too easy for gLM's and so they rebalanced the datasets that they constructed to have an accuracy of between 0.3 and 0.9, to provide a more challenging benchmark for gLMs. The epigenetic marks prediction tasks are based on the same dataset as the Nucleotide Transformer Tasks version 1, and so we do not provide repeated results for these datasets, instead providing results for the epigenetic marks prediction tasks in NTTv2 because these datasets have been curated and balanced and are more robust than the originals. They also introduce tasks of human transcription factor prediction, mouse transcription factor prediction, and a challenging covid variant classification task with nine classes.

Promoter Detection (Human) The data for these three datasets came from the Eukaryotic Promoter Dataset (EPDnew), the same primary source as the Nucleotide Transformer benchmark [3].

Core Promoter Detection (Human) Similar to the Promoter Detection dataset, but the window of nucleotides included are -34 and +35 from the TSS, which should make this a more challenging task than proximal promoter prediction.

Splice Site Prediction (Human) The data for this task was originally compiled by Wang et al. 2019. The dataset consists of positive and negative examples of donor, acceptor and non-splice sites that were extracted from Ensemble GRCh38. This is the same source as the original splice_sites_all task from Dalla-Torre et al. 2023 [3], but the dataset was reconstructed by Zhou et al. 2024 with more adversarial non-cannonical splice site examples to make it more challenging.

Transcription Factor Binding Site Prediction (Human) This dataset was constructed by downloading 690 ENCODE ChIP-seq experiments from 2012 and extracted a 101 bp region around the center of each peak for the true positive class and extracted sequences that were not overlapping of the same length for the negative class. Five datasets were randomly constructed from this set and they were curated heuristically so that the accuracy range would have an F1 score between 0.5 and 0.95.

Transcription Factor Binding Site Prediction (Mouse) Similar to the TFBS prediction for human DNA, but mouse ENCODE ChIP-seq data came from Stamatoyannopoulos et al. 2012, and negative examples were created using dinucleotide shuffling while preserving relative frequencies.

Covid Variant Prediction (Virus) This dataset was compiled by Zhou et al. [15]. They downloaded viral genomes from EpiCov database from GISAID. They extract 1000 bp segments from each genome and classify nine variants of the virus, Alpha, Beta, Delta, Eta, Gamma, Iota, Kappa, Lambda and Zeta.

Enhancer promoter interaction (Human) This dataset was compiled by Zhou et al. [15] and discerns the interactions between promoters and enhancers.

References

1. Robin Andersson, Claudia Gebhard, Irene Miguel-Escalada, Ilka Hoof, Jette Bornholdt, Mette Boyd, Yun Chen, Xiaobei Zhao, Christian Schmidl, Takahiro Suzuki, Evgenia Ntini, Erik Arner, Eivind Valen, Kang Li, Lucia Schwarzfischer, Dagmar Glatz, Johanna Raithel, Berit Lilje, Nicolas Rapin, Frederik Otzen Bagger, Mette Jørgensen, Peter Refsing Andersen, Nicolas Bertin, Owen Rackham, A. Maxwell Burroughs, J. Kenneth Baillie, Yuri Ishizu, Yuri Shimizu, Erina Furuhata, Shiori Maeda, Yutaka Negishi, Christopher J. Mungall, Terrence F. Meehan, Timo Lassmann, Masayoshi Itoh, Hideya Kawaji, Naoto Kondo, Jun Kawai, Andreas Lennartsson, Carsten O. Daub, Peter Heutink, David A. Hume, Torben Heick Jensen, Harukazu Suzuki, Yoshihide Hayashizaki, Ferenc Müller, Alistair R. R. Forrest, Piero Carninci, Michael Rehli, and Albin Sandelin. An atlas of active enhancers across human cell types and tissues. *Nature*, 507(7493):455–461, March 2014.
2. Dikla Cohn, Or Zuk, and Tommy Kaplan. Enhancer identification using transfer and adversarial deep learning of DNA sequences, February 2018. Pages: 264200 Section: New Results.
3. Hugo Dalla-Torre, Liam Gonzalez, Javier Mendoza-Revilla, Nicolas Lopez Carranza, Adam Henryk Grzywaczewski, Francesco Oteri, Christian Dallago, Evan Trop, Bernardo P. de Almeida, Hassan Sirelkhatim, Guillaume Richard, Marcin Skwark, Karim Beguir, Marie Lopez, and Thomas Pierrot. The Nucleotide Transformer: building and evaluating robust foundation models for human genomics, October 2024. bioRxiv: 10.1101/2023.01.11.523679.
4. René Dreos, Giovanna Ambrosini, Rouayda Cavin Périer, and Philipp Bucher. EPD and EPDnew, high-quality promoter resources in the next-generation sequencing era. *Nucleic Acids Research*, 41(Database issue):D157–164, January 2013.
5. Katarína Grešová, Vlastimil Martinek, David Čechák, Petr Šimeček, and Panagiotis Alexiou. Genomic benchmarks: a collection of datasets for genomic sequence classification. *BMC Genomic Data*, 24(1):25, May 2023.
6. Kevin L Howe, Premanand Achuthan, James Allen, Jamie Allen, Jorge Alvarez-Jarreta, M Ridwan Amode, Irina M Armean, Andrey G Azov, Ruth Bennett, Jyothish Bhai, Konstantinos Billis, Sanjay Boddu, Mehrnaz Charkhchi, Carla Cummins, Luca Da Rin Fioretto, Claire Davidson, Kamalkumar Dodiya, Bilal El Houdaigui, Reham Fatima, Astrid Gall, Carlos Garcia Giron, Tiago Grego, Cristina Guijarro-Clarke, Leanne Haggerty, Anmol Hemrom, Thibaut Hourlier, Osagie G Izuogu, Thomas Juettemann, Vinay

Kaikala, Mike Kay, Ilias Lavidas, Tuan Le, Diana Lemos, Jose Gonzalez Martinez, José Carlos Marugán, Thomas Maurel, Aoife C McMahon, Shamika Mohanan, Benjamin Moore, Matthieu Muffato, Denye N Oheh, Dimitrios Paraschas, Anne Parker, Andrew Parton, Irina Prosovetskaia, Manoj P Sakthivel, Ahamed I Abdul Salam, Bianca M Schmitt, Helen Schuilenburg, Dan Sheppard, Emily Steed, Michal Szpak, Marek Szuba, Kieron Taylor, Anja Thormann, Glen Threadgold, Brandon Walts, Andrea Winterbottom, Marc Chakiachvili, Ameya Chaubal, Nishadi De Silva, Bethany Flint, Adam Frankish, Sarah E Hunt, Garth R IIsley, Nick Langridge, Jane E Loveland, Fergal J Martin, Jonathan M Mudge, Joanella Morales, Emily Perry, Magali Ruffier, John Tate, David Thybert, Stephen J Trevanion, Fiona Cunningham, Andrew D Yates, Daniel R Zerbino, and Paul Flicek. Ensembl 2021. *Nucleic Acids Research*, 49(D1):D884–D891, January 2021.

7. Takahiro Ishii, Ken-ichi Yoshida, Goro Terai, Yasutaro Fujita, and Kenta Nakai. DBTBS: a database of Bacillus subtilis promoters and transcription factors. *Nucleic Acids Research*, 29(1):278–280, January 2001.
8. Y. Luo, B.C. Hitz, I. Gabdank, J.A. Hilton, M.S. Kagda, B. Lam, Z. Myers, P. Sud, J. Jou, K. Lin, U.K. Baymuradov, K. Graham, C. Litton, S.R. Miyasato, J.S. Strattan, O. Jolanki, J.W. Lee, F.Y. Tanaka, P. Adenekan, E. O'Neill, and J.M. Cherry. New developments on the Encyclopedia of DNA Elements (ENCODE) data portal. *Nucleic Acids Research*, 48(D1):D882–D889, January 2020.
9. Rouaïda Cavin Périer, Viviane Praz, Thomas Junier, Claude Bonnard, and Philipp Bucher. The Eukaryotic Promoter Database (EPD). *Nucleic Acids Research*, 28(1):302–303, January 2000.
10. Yair Schiff, Chia-Hsiang Kao, Aaron Gokaslan, Tri Dao, Albert Gu, and Volodymyr Kuleshov. Caduceus: Bi-directional equivariant long-range DNA sequence modeling. *arXiv preprint arXiv:2403.03234*, 2024.
11. Petr Simecek. PseudoDNA Generator, July 2020. https://github.com/simecek/ PseudoDNA_Generator.
12. Ramzan Kh Umarov and Victor V. Solovyev. Recognition of prokaryotic and eukaryotic promoters using convolutional deep learning neural networks. *PLoS One*, 12(2):e0171410, February 2017.
13. Daniel R. Zerbino, Steven P. Wilder, Nathan Johnson, Thomas Juettemann, and Paul R. Flicek. The ensembl regulatory build. *Genome Biology*, 16(1):56, March 2015.
14. Jingjing Zhai, Aaron Gokaslan, Yair Schiff, Ana Berthel, Zong-Yan Liu, Wei-Yun Lai, Zachary R. Miller, Armin Scheben, Michelle C. Stitzer, M. Cinta Romay, Edward S. Buckler, and Volodymyr Kuleshov. Cross-species modeling of plant genomes at single nucleotide resolution using a pre-trained DNA language model. *bioRxiv: The Preprint Server for Biology*, page 2024.06.04.596709, August 2024.
15. Zhihan Zhou, Yanrong Ji, Weijian Li, Pratik Dutta, Ramana Davuluri, and Han Liu. DNABERT-2: efficient foundation model and benchmark for multi-species genome, June 2023. arXiv: 2306.15006.
